# Strengths and Weaknesses of Light Transmission Aggregometry in Diagnosing Hereditary Platelet Function Disorders

**DOI:** 10.3390/jcm9030763

**Published:** 2020-03-12

**Authors:** Marie-Christine Alessi, Pierre Sié, Bernard Payrastre

**Affiliations:** 1 Aix Marseille Univ, Inserm, Inrae, C2VN, 13385 Marseille CEDEX, France; 2 CHU de Toulouse, Laboratoire d’Hématologie, 31059 Toulouse CEDEX, France; sie.p@chu-toulouse.fr; 3 Inserm U1048, I2MC et Université Paul Sabatier, 31024 Toulouse CEDEX, France; bernard.payrastre@inserm.fr

**Keywords:** platelets, light transmission aggregometry, inherited platelet disorders, diagnosis

## Abstract

Hereditary defects in platelet function are responsible for sometimes severe mucocutaneous hemorrhages. They are a heterogeneous group of abnormalities whose first-line diagnosis typically involves interpreting the results of in vitro light transmission aggregometry (LTA) traces. Interpretation of LTA is challenging. LTA is usually performed in specialized laboratories with expertise in platelet pathophysiology. This review updates knowledge on LTA, describing the various platelet aggregation profiles typical of hereditary platelet disorders to guide the physician in the diagnosis of functional platelet disorders.

## 1. Introduction

In vitro light transmission platelet aggregation (LTA) was described 50 years ago [1,2,3]. A recent survey of diagnostic laboratories on inherited platelet dysfunctions found that most laboratories perform LTA. This test has good performance characteristics and detects abnormalities associated with increased bleeding in a significant proportion of individuals referred for platelet function disorder (PFD) investigations [4]. LTA is typically used as a first-line test to guide diagnosis of PFD. 

The principle of the test is the photometric measurement of platelet rich plasma (PRP) secondary to platelet aggregation induced by relevant activators. In vitro platelet aggregation is the consequence of platelet activation induced by various soluble activators. It involves specific membrane receptors, downstream signaling pathways, and amplification pathways that vary depending on the type and amount of the activator used. Ultimately, the activated membrane receptor complex, glycoprotein (GP) IIb/IIIa (integrin αIIbβ3), binds fibrinogen and in this way provides bridges between platelets. Via the GPIIbIIIa/fibrinogen/GPIIbIIIa bridges, platelets form aggregates. Aggregation of platelets makes cell suspension less turbid (decreases optical density) because the number of light-scattering particles in the suspension decreases. The kinetics of the response provides quantitative assessment of platelet aggregation. The slope of the curve, the peak extent (%), and the latency time (lag phase) are often automatically measured. In addition, the initial shape change, mono- or biphasic aggregation waves, and the stability of the aggregates (reversibility) can be visually assessed. Defects in platelet aggregation may refer to a reduced response to a single activator, but in most cases, the diagnosis process should interpret reduced responses to multiple activators, since the activation of signaling and amplification pathways is shared among them. 

This review seeks to provide a practical guide to the interpretation of LTA. We first summarize the main pathways of platelet activation and the conditions under which the test has to be carried out. We then describe the aggregation profiles of the most frequently encountered platelet disorders with established molecular defects. Lastly, we present the other platelet disorders described in the literature, whose molecular defects remain unknown. 

## 2. The Main Pathways of Platelet Activation

**Figure 1:**
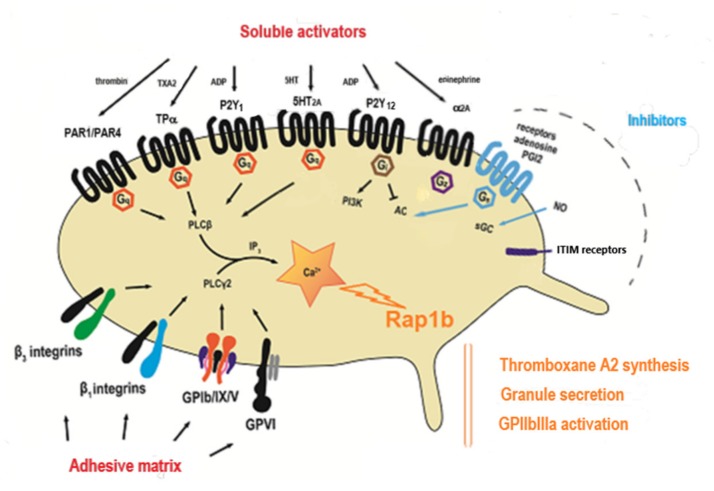
Primary platelet activation pathways. Gq, G protein-containing sub-unit αq; Gi, G protein-containing sub-unit αi; Gz, G protein-containing sub-unit αz; Gs, G protein-containing sub-unit αs; ADP, adenosine diphosphate; TXA_2_, thromboxane A_2_; TP, TXA_2_ receptor; PAR, protease-activated receptor; P2Y_1_/P2Y_12_, purinergic receptors; PLC-β, phospholipase C-β; PLC-γ, phospholipase C-γ; PI-3-K, phosphoinositide-3-kinase; Akt, protein kinase B; AC, adenylate cyclase; sGC, soluble adenylate cyclase; Rap1b, Ras-related protein Rap-1b; 5HT, 5 hydroxytryptamine (or serotonin); 5HT_2A_, Serotonin 5-HT_2A_ receptors.

Platelet activation pathways can be schematically characterized as those involved in platelet adhesion induced by contact with the injured vessel wall, and those that contribute to the amplification of platelet activation induced by soluble mediators released or produced on platelet activation. The adhesion phenomena mainly involve adhesive proteins such as collagen, fibronectin, Willebrand factor, laminins, fibrinogen (as integrin-like membrane glycoprotein ligands), and immunoreceptors with Immunoreceptor Tyrosine-based Activation Motif (ITAM motifs) (such as GPVI associated with the Fc γ receptor) or leucine-rich motifs (such as GPIb-V-IX) which activate downstream signaling pathways linked to Src kinases. Through a series of effector recruitments and substrate phosphorylations, activation of phospholipase Cγ2 ensues. Collagen is used to explore the GPVI pathway and ristocetin to evaluate the GPIb pathway. Ristocetin is an antibiotic no longer used in clinical practice owing to side effects such as the development of thrombocytopenia induced by platelet agglutination. This antibiotic causes the binding of Willebrand factor to the GPIb. It is currently used exclusively in vitro as a platelet agglutination activator to identify defects of the Willebrand/GPIb axis. Soluble mediators (primarily adenosine diphosphate (ADP), thromboxane A_2_ (TXA_2_), serotonin also known as 5 hydroxytryptamine (5HT), and thrombin or thrombin-like peptides (TRAP)) activate G Protein Coupled Receptors (GPCRs), seven-membrane receptors that are molecular switches for signal transduction. These receptors (mainly purinergic receptors P2Y_1_, and P2Y_12_, thromboxane A_2_ receptor TPα, and protease-activated receptors PAR1 and PAR4) initiate intracellular signaling pathways through the phospholipase Cβ isoform (Figure 1). 

**Figure 2:**
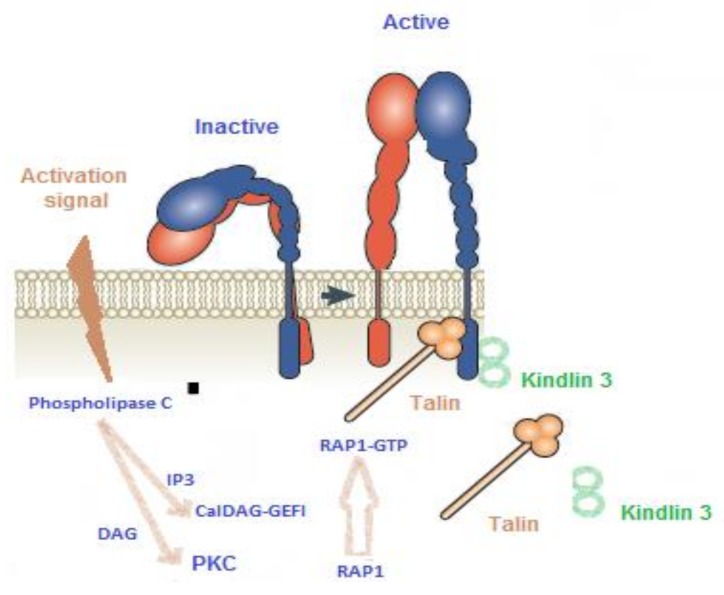
Schematic representation of the signaling pathways involved in the activation of integrins by CalDAG-GEFI, a GDP/GTP exchange factor specific to small G proteins, Rap. The interaction between Rap1-GTP and Rap1-interacting molecule (RIAM) (not shown) stimulates talin binding to beta-integrin. Kindlin 3 binds to the cytoplasmic region of beta-integrin, allowing its clustering and transition from an inactive to an active form. DAG, 1,2-diacyl-glycerol; PKC, protein kinases C; IP3, inositol-1,4,5-trisphosphate.

Phospholipase C hydrolyzes phosphoinositide-4,5-bisphosphate (PIP2) to inositol-1,4,5-trisphosphate (IP3) and 1,2-diacyl-glycerol (DAG). IP3 induces the release of Ca^2+^ from intracellular stores in the platelet cytoplasm and DAG activates protein kinases C (PKCs), which results in platelet-sustained granule secretion, subsequent ADP release, TXA_2_ synthesis, and GPIIbIIIa receptor activation (Figure 2). 

These Ca^2^+-sensitive and PKC pathways have been shown to act separately but synergistically in the activation of αIIbβ3 integrin [5]. The Ca^2^+-sensitive pathway involves the rapid activation of CalDAG-GEF1, a guanine nucleotide exchange factor that activates Rap1b, a GTP-binding protein that possesses intrinsic GTPase activity, an essential signaling node in “inside-out” activation of the integrin GPIIbIIIa. The PKC pathway is independent of CalDAG-GEFI and leads to slower but sustained Rap1 activation (Figure 2). These events induce cytoskeleton re-arrangements, extrusion of platelet granules, and conversion of integrin GPIIbIIIa into a high-affinity state (“inside-out” signaling). Finally, ligand interaction with GPIIbIIIa (mainly fibrinogen) bridges platelets together and triggers “outside-in” signaling, which orchestrates cytoskeletal rearrangements for platelet spreading and clot stabilization. Understanding the platelet signaling machinery involved in thrombus formation is necessary to identify potential targets for platelet function disorders.

## 3. Carrying Out LTA

LTA is sensitive to the technical conditions in which the assay is performed, particularly the pre-analytic steps. The British Society of Haematology published guidelines in 1988 [6], and Hayward et al. presented a North America Consensus in 2010 [7]. Recommendations were published by the “platelet” subcommittee of the International Society on Thrombosis and Haemostasis (ISTH/SSC) in 2013 [8] followed by guidelines from the Permanent Paediatric Committee of the Society of Thrombosis and Haemostasis Research (GTH e. V.) [9]. These documents were recently implemented with the publication of a diagnostic guide for hereditary platelet disorders [10]. Blood samples should be drawn with minimal or no venostasis, using an appropriate needle, into polypropylene or siliconized glass tubes. PRP should be prepared by centrifuging blood samples at 200× *g* for 10 min at room temperature. Among the most important recommendations are the maintenance of a PRP platelet count above 150 × 10^9^/L. Below this threshold, interpretation of the results will be haphazard. We note that platelets may differ regarding the relative proportion of large and small platelets. Increasing g-forces progressively depletes PRP of its larger platelets. LTA results should therefore be assessed with caution if platelet size has not been evaluated. The recommendation to avoid using a rotor brake is based on expert opinion and needs to be demonstrated [11]. Grossly hemolysed and lipemic samples should be discarded. It is not recommended to adjust platelet count except in cases of extremely high platelet count. PRP should be allowed to stand at room temperature for 15 min before testing, although a 30 min resting period has been proposed in other guidelines [12], and analysis should be completed within a maximum 4 h after blood sampling and sooner (2 h) once the PRP has been prepared (personal appraisal). Finally, it is important to keep in mind that several aggregation defects cannot necessarily be reproduced along successive tests, because pathophysiological variations or unreported intake of medications or xenobiotic agents can modify platelet behavior. It is therefore essential to confirm the results at least once, on distant sampling, especially when the observed defects are moderate.

The platelet aggregation profiles vary according to the activator used. For some activators (e.g., collagen, arachidonic acid (AA), ristocetin, and thrombin receptor-activating peptides (TRAP)), the concentration effect curve is characterized by an acute slope with a threshold effect leading to a strong variation in the intensity of platelet aggregation for a small modification of the activator concentration. Therefore, appropriate concentrations of activators must be chosen to limit excessive inter–individual variability and interpretation difficulties. In our hands, the inter–individual coefficients of variation (CV) for maximal intensity calculated from 60 healthy volunteers, range between 6.7% and 11.4% using the concentrations recommended by the ISTH guidance document (collagen 2 µg/mL, AA 1 mM, ristocetin 1.2 mg/mL, and epinephrine 5 µM) [7]. Low ADP concentration (2 µM) is associated with higher variability (CV = 17.4%). In addition, commercially available aggregometers and reagents do not all have the same technical specifications (test volume, activator source and characteristics, etc.), which limits interlaboratory comparison. External quality controls could help standardize LTA but sending reference PRP samples is not currently conceivable owing to technical limitations. As an effort to improve LTA quality control, the North American Specialized Coagulation Laboratory Association introduces biologists to the interpretation of normal or pathological LTA traces. Another way to improve quality is to standardize the activators used. Marketed reagents have seldom been compared in the literature. The THROMKID-Plus study group initiated a first LTA inter-laboratory trial in Germany and Austria by sending stable activators (ADP, AA, collagen type I, TRAP-6, and ristocetin) to 14 laboratories [13]. The final concentrations and the pre-analytical issues were selected for the LTA according to the recommendations of the ISTH/SSC [8]. All the laboratories used fresh PRP from a locally recruited healthy donor. The participating laboratories obtained similar maximum platelet aggregation values for all the tested activators. When the participating laboratories tested their own activators and concentrations with the same PRP as that already used for the shipped activators, high inter-laboratory variability was observed, arguing for own reference intervals as proposed by the ISTH [8] and the feasibility of activator shipment as a suitable inter-laboratory survey of LTA. As part of the ISTH/SSC, an international, multi-center study has been set up to evaluate the extent of variability among commercial and in-house activators. This study also includes reference activators. It will provide evidence to support setting reference activators to standardize platelet aggregation.

## 4. Hereditary Platelet Disorders with Known Molecular Defects

We will first discuss the absence of aggregation in response to multiple activators, followed by reduced aggregation in response to multiple activators (except for ristocetin, which explores the Willebrand/GPIb axis) and then defects in the response to a single activator. 

### 4.1. Absence of Aggregation in Response to Multiple Activators Except for Ristocetin

This diagnosis is made when no aggregation occurs in response to multiple activators at low or high concentrations, particularly protease-activated receptor (PAR) activators (thrombin or TRAP), while the response to ristocetin is maintained, although it is sometimes reversible or even cyclic [14]. The hemorrhagic syndrome is typically severe. The defect is associated with an absence or marked reduction of fibrinogen binding to its platelet receptor, GpIIb/IIIa.

Quantitative deficiency in GpIIb/IIIa on the platelet surface constitutes the Glanzmann thrombasthenia, an autosomal recessive disorder whose diagnosis is most often confirmed by the complete loss of GPIIb/IIIa expression on the platelet surface as assessed by flow cytometry [15]. Simple immunofluorescence labeling and imaging may suffice if a flow cytometer is unavailable.

Absence of platelet aggregation in response to all activators with preservation of GpIIb/IIIa expression suggests either a variant of Glanzmann thrombasthenia, or a kindlin 3 deficiency.

A qualitative GpIIb/IIIa abnormality leading to defective activation that prevents fibrinogen binding constitutes the Glanzmann thrombasthenia variant. In this case, diagnosis can be made by the inability of stimulated platelets to bind either PAC-1, a monoclonal antibody that specifically recognizes the activated form of the GpIIb/IIIa, or fluorescently-labeled fibrinogen. As part of a differential diagnosis, the presence of receptor-blocking autoantibodies inducing a thrombasthenia-like state [16,17] needs to be excluded in isolated cases with no family history.

A kindlin 3 (official gene symbol *FERMT3*) deficiency causes autosomal recessive type III leukocyte adhesion deficiency (LADIII). Kindlin-3 associates with the actin cytoskeleton and is essential for proper GpIIb/IIIa activation [18] (Figure 2). 

**Figure 3:**
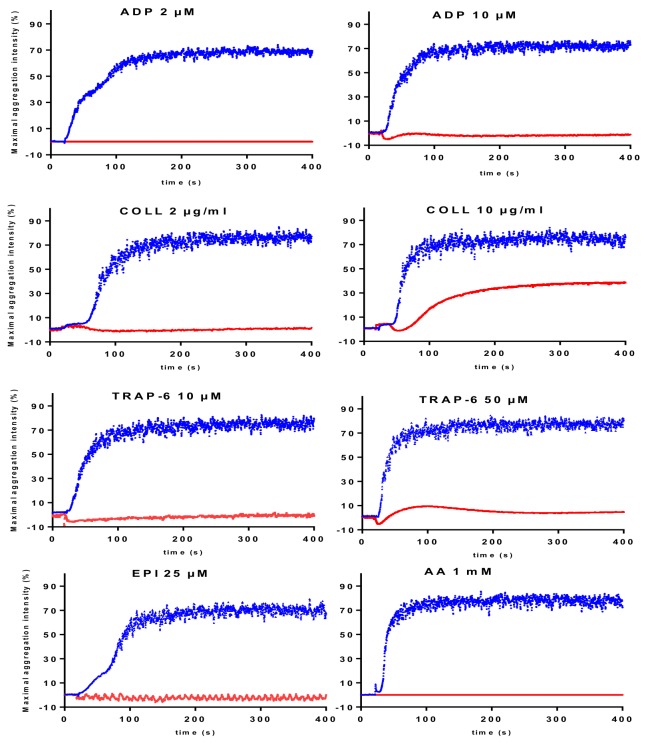
LTA in response to different activators (ADP, collagen (Coll), thrombin or thrombin-like peptides (TRAP-6), epinephrine (EPI) and arachidonic acid (AA)) at low and high concentrations in a patient (red line) carrying a homozygous mutation in the *FERMT3* gene leading to kindlin-3 deficiency [9] compared with a healthy individual (blue line). Aggregation profiles are obtained using a TA-8V aggregometer from SD Innovation and reagents from Agro-bio.

Kindlin-3-deficient platelets show almost no aggregation in response to any activators except ristocetin. Residual aggregation (≈ 30% of normal) can persist at high concentrations of collagen or TRAP (Figure 3) [18]. 

Adding Mn^2+^ forces the GpIIb/IIIa to adopt an active conformation, independently of “inside-out signaling”, and thus of kindlin-3 and subsequently corrects, at least partially, the aggregation defect [18]. This platelet function defect is accompanied by immune deficiency, with primary signs including hyperleukocytosis and recurrent infections, which distinguishes LADIII from the Glanzmann variant.

### 4.2. Reduced LTA in Response to Multiple Activators

This corresponds to the most frequent situation. The decrease in the response to several activators is variable in intensity according to the origin of the defect and the type and concentration of the activator used.

#### 4.2.1. CalDAG-GEFI Deficiency

**Figure 4:**
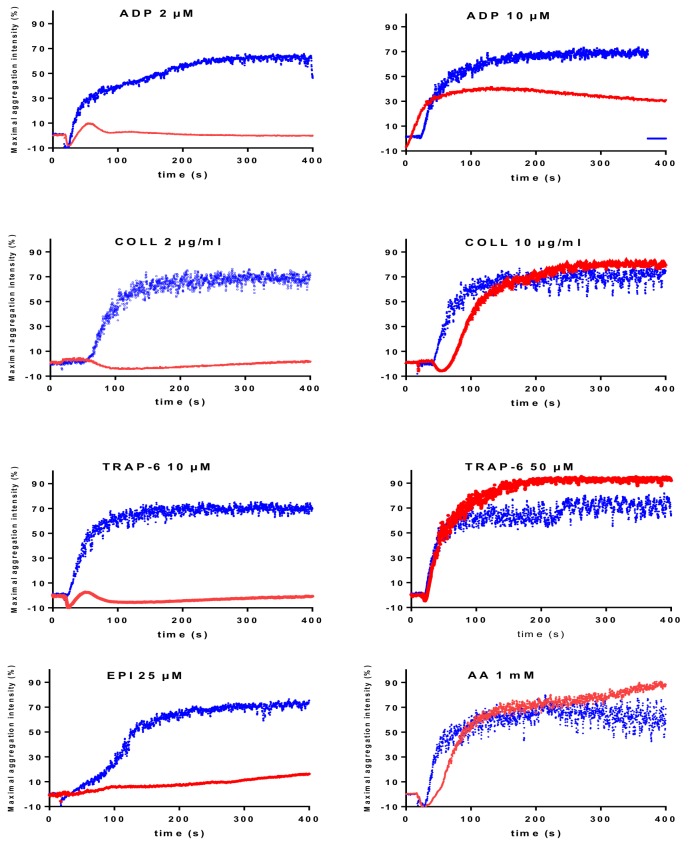
LTA in response to several activators (ADP, collagen (Coll), TRAP-6, epinephrine (EPI) and arachidonic acid (AA) at different concentrations in a patient (redline) exhibiting a homozygous functional deficiency in CalDAG-GEFI [17] compared with a healthy individual (blue line). Aggregation profiles are data obtained using a TA-8V aggregometer from SD Innovation and reagents from Agro-bio.

CalDAG-GEFI (or RasGRP2) is a nucleotide exchange factor for the monomeric GTPase Rap1, involved in the integrin “inside-out” signaling pathway (Figure 2). We reported the first human cases of CalDAG-GEFI defect in a French family carrying a mutation in the CalDAG-GEFI coding gene, *RASGRP2*. The mutation is in a region coding for a domain of the protein required for the interaction with Rap1b [19]. Since then, more than 20 families carrying *RASGRP2* mutations have been identified and described in the literature. Owing to the importance of CalDAG-GEFI for the rapid, but reversible, activation of Rap1, secondary to an increase in cytoplasmic Ca^2+^ concentration, the disorder is associated with reduced platelet response to multiple activators. Platelet aggregation issubstantially reduced when the platelets are stimulated by low concentrations of several activators (2 μM ADP, 10 μM TRAP, and 2 μg/mL collagen). A decrease in maximal intensity was present with higher concentrations of ADP (5–10 µM) and epinephrine (5–25 µM). However, the loss of CalDAG-GEFI is compensated for if high concentrations of activators are used, such as TRAP (50 μM), collagen (10 μg/mL), AA (1.5 mM), or even ADP (50–100 μM) (Figure 4), or by direct activation of protein kinase C (PKC) by phorbol 12-myristate 13-acetate (PMA). The response to ristocetin (1.2 mg/mL) is unaffected.

#### 4.2.2. Ephrin B2-Related Platelet Disorder

In 2018, Berrou et al. [20] identified a new inherited platelet bleeding disorder affecting the *EPHB2* gene in two siblings from a consanguineous family. *EPHB2* encodes the ephrin transmembrane receptor B2 tyrosine kinases (EPHB2). Both patients exhibited recurrent bleeding and normal or marginally decreased platelet counts. Platelet aggregation was absent in response to collagen and was impaired in response to 10 µM ADP, 1.5 mM AA, and 5 µM U46619. Aggregation induced by 1.5 mg/mL ristocetin and 25 µM PAR1-AP was normal. Aggregation of washed platelets induced by various concentrations of ADP (10 and 20 μM), thrombin (25 and 50 mU/mL), and 50 μM PAR4-AP (a selective proteinase-activated receptor 4 specific activator peptide) was strongly impaired. It is suggested that EPHB2 plays a relevant role at a distal level of G-protein coupled receptors. The fact that the discovered EPHB2 variant affects the phosphorylation of Lyn and Src induced by the GP VI activator, convulxin, and PAR4-AP, suggests that EPHB2 activates phosphatases involved in the dephosphorylation of the inhibitory tyrosine residue of Src required for its activation. The abnormalities for EPHB2 were not limited to platelet function and also concerned platelet morphology, with occasional thin and abnormally long platelets. The discovery of new cases will bring a better understanding of this recently discovered platelet dysfunction.

#### 4.2.3. Abnormality of the Amplification Pathway Mediated by ADP

Platelets express multiple purinergic receptors: two are G-protein coupled ADP receptors (P2Y_1_ and P2Y_12_); and a third is an ATP receptor, P2X_1_, which functions as a calcium channel [21]. ADP-induced platelet aggregation requires both the P2Y_1_/Gq pathway, considered as a “starter” pathway, and the P2Y_12_/Gi pathway, which is an amplification pathway (Figure 1). 

The P2Y_1_ receptor is coupled to Gαq and triggers the mobilization of calcium from internal stores. It is absolutely required for ADP-induced platelet aggregation. P2Y_1_ activation is characterized by a platelet shape change visible on the trace at the foot of the aggregation curve. The P2X_1_ receptor binds ATP; it is not enough to trigger aggregation per se but amplifies the P2Y_1_/Gq stimulation signal and reinforces aggregation induced by ADP and other activators. The P2Y_12_ receptor is responsible for completion of the platelet aggregation response to ADP initiated by P2Y_1_, and for the ADP-dependent amplification of platelet aggregation induced by other activators. Its activation inhibits the GPIIbIIIa activation negative feedback pathway, mediated by adenosine monophosphate (AMPc). AMPc is produced by adenylate cyclase (AC) (Figure 1) when activated by a signal through the endothelial prostacyclin (PGI_2_), prostaglandin 1 (PGE_1_) or adenosine-specific receptors present at the platelet surface, or directly by forskolin. This feedback mechanism is usually not explored as part of the diagnosis and requires the implementation of appropriate aggregation-inhibition studies based on the addition of a Gs activator prior to induction of platelet aggregation. 

The ADP contained and secreted by platelet δ (dense) granules acts as an amplifier of platelet activation induced by low concentrations of most activators besides ADP itself, including collagen, AA, U46619, and PAR receptor agonists. A defect in the ADP pathway, whatever its cause (P2Y_12_ receptor or ADP/ATP secretion defects), will therefore reduce the response to sub-optimal concentrations of most of the activators by interrupting this amplification loop. This amplification is no longer essential if high concentrations of activators are used, meaning that on high concentration activator stimulation, some defects (mainly secretion defects) may remain unidentified.

The LTA profile in response to ADP depends on the concentration of ADP used and may involve the role of P2Y_12_ receptors in potentiation of platelet secretion. At low concentrations (1–2 μM), the aggregation is reversible. Intermediate concentrations (2–4 μM) give rise to what is known as the double wave phenomenon (aggregation followed by initial disaggregation, then complete, stable aggregation). At high concentrations (≥5–10 μM), the aggregation is stable. 

Defects in aggregation induced by ADP can have multiple causes. However, responses to different ADP concentrations and a defective response to other activators will give some idea of its origin.

- P2Y_12_ Receptor Abnormality

In patients carrying deleterious homozygous mutations, a defective response to ADP is often total, even at very high concentrations of ADP (100 μM) with a markedly reduced and rapidly reversible aggregation [22,23,24,25]. The severity depends not only on the number, but also on the quality of the receptors at the platelet surface. The p.His187Gln variant that impairs conformational mobility of the receptor causes no change in its expression but decreases its affinity for the ligand [23]. The p.Arg122Cys substitution reduces surface P2Y_12_ expression. This is related to an activator-independent internalization followed by P2Y_12_ receptor trafficking to lysosomes [24]. The activator-induced platelet shape change is not affected, because it is dependent on the P2Y_1_ receptor. However, LTA is reduced in response to low concentrations of most activators [25], highlighting the amplifying role of ADP under weakly stimulating conditions. This phenomenon is not or only weakly evident at high concentrations of collagen (>10 μg/mL) or TRAP (generally 50 μM). A slight reduction in aggregation in response to epinephrine (10 μM) can be seen. The LTA induced by AA is also moderately reduced. The weak repercussion on epinephrine and AA-induced responses is probably because the concentrations used are quite high, preventing the amplifying role of secretion from being visualized. The half maximal effective concentration (EC50) of epinephrine and AA are around 0.1 µM and 0.4 mM respectively (personal data).

Patients carrying heterozygous mutations in *P2RY12* will generally exhibit an intermediate clinical picture with reduced, reversible ADP-induced platelet aggregation that improves with high concentrations of ADP [26,27,28,29,30,31,32,33,34]. It could be speculated that the impact on LTA will depend on the number and quality of P2Y_12_ receptors present on the platelet surface. Heterozygous p.Lys147Glu substitution reduces the ability of platelets to bind ADP by half, and the traces obtained in response to stimulation with concentrations of ADP higher than 30 μM are almost normal [26]. In the case of a heterozygous deletion of two nucleotides within the coding region, at amino acid 240 [28,29], RT–PCR products demonstrated that the *P2RY12* transcripts derived from the mutant allele with no wild-type product. The mechanism by which expression of the wild-type allele is repressed is, however, not clear. Other heterozygous mutations, such as the p.Pro341Ala variation, whose ADP-binding capacity is unaffected, are not associated with aggregation abnormality. Platelets from the subject carrying this mutation showed sustained aggregation and dense granule secretion, which were similar to that of control platelets, in response to a broad range of ADP concentrations (1–100 μM) [30]. The analysis of the functional consequence of the mutation is therefore based on a more sensitive method that evaluates a loss in adenylate cyclase inhibition even at very low concentrations of ADP (0.1 μM) when pre-stimulated with prostaglandin E2 (PGE_2_) [30] or forskolin or, low vasodilator-stimulated phosphoprotein (VASP) dephosphorylation in response to ADP after treatment with prostaglandin E1 (PGE_1_) [35].

- Defects in δ Granule Secretion

Chediak–Higashi disease [36] and Hermansky–Pudlak diseases [37] are autosomal recessive disorders characterized by a deficiency in δ granules and are associated with mutations in *LYST* or *HPS* genes, respectively. These conditions are suggested by the presence of oculocutaneous albinism although in some cases (HPS5 and HPS6), skin albinism may go unnoticed and the picture is limited to ocular albinism. 

Besides these syndromes, δ granule deficiency (δ-storage pool disease) may be observed in association with constitutional thrombocytopenia without albinism. Delta granule deficiency has been described in FDP-AML- [38], *FLI-1-* [39] along with *FLNA-* [40] or *GATA1-* [41] related thrombocytopenia. It may also be observed in acquired hematopoietic diseases, notably in patients with myelodysplastic and myeloproliferative disorders [42]. 

These disorders are characterized by a reduction in the amplifying roles of granular secretion. LTA is sometimes impaired. In one study, 23% of 106 patients presented normal aggregation to ADP, epinephrine, and collagen [43]. This is presumably due to defect variability or the implementation of compensation pathways. The LTA profiles are similar to those seen with P2Y_12_ deficiency. The responses to low concentrations of most of the activators are reduced [44], but the defective response to 10 μM ADP is usually moderate [44]. 

In suspected cases, complementary methods should be applied, particularly measurement of the content and secretion of δ granules (ATP and 5HT (or serotonin) concentrations, mepacrine tests, membrane expression of CD63, and whole-mount transmission electron microscope enumeration of dense granules in fixed platelets).

#### 4.2.4. Abnormality of the Amplification Pathway Mediated by Thromboxane A2 (TXA_2_)

When exogenous AA is added to PRP, it is rapidly transformed into TXA_2_ through the activity of platelet-derived cyclo-oxygenase 1 (COX-1), also known as prostaglandin G/H synthase 1 (PGHS1). This transformation also occurs when endogenous AA contained in membrane phospholipids is released through the activity of phospholipase A during platelet activation. The TXA_2_ produced by the transformation of endogenous or exogenous AA exerts autocrine and paracrine effects via a specific receptor (TPα) coupled to Gq (Figure 1). This pathway represents an amplification mechanism required for aggregation that occurs at low concentrations of many activators. Abnormality of the amplification pathway mediated by TXA_2_ is most often due to an autosomal dominant variation in the gene coding the TPα receptor, *TBXA2R,* but can also correspond to a reduced conversion of exogenous AA to TXA_2_. The use of TXA_2_ analogs, such as U46619 or STA2, as activators, and measurement of Thromboxane B2 (TXB_2_) levels (stable metabolite of TXA_2_) can help distinguish between these two mechanisms.

- Variation in Receptor TPα

This is seen not only as a defective response to AA (0.5–1.5 mM) [45] but also by the absence of a second wave in response to epinephrine and a reduced LTA at low concentrations (2 μg/mL) of collagen and ADP [46,47]. The aggregation induced by U46619 (1–3 µM) or STA2 is reduced [45,46,47,48,49] but can be corrected in some cases if the concentration of U46619 is increased (10 µM) [38]. Reduced surface receptor expression [46] or a poor Gq protein coupling without impaired TXA_2_ binding to the TPα receptor [48] have been implicated.

- Defect in the Conversion of AA to TXA_2_

In this case the LTA induced by TXA_2_ analogs is normal, and serum TXB_2_ levels are very low. This type of defect should suggest first-line treatment with COX-1 inhibitors such as non-steroidal anti-inflammatory drugs including aspirin. Mutations in *TBXAS1*, which encodes a thromboxane synthase, have been identified in patients with the rare autosomal recessive Ghosal hematodiaphyseal dysplasia (increased diaphyseal bone density and a regenerative corticosteroid-sensitive anemia), associated with defective response to AA primarily at very low concentrations, while the aggregation induced by U46619 is normal [50]. To date, variations in the genes coding phospholipase A2 or COX-1 involved in TXA_2_ production have not been described.

#### 4.2.5. ARC Syndrome

Arthrogryposis renal dysfunction and cholestasis (ARC) syndrome is a very rare lethal infantile disease caused by loss-of-function mutations in genes coding the vacuolar protein sorting 33 homolog B (*VPS33B*) or *VPS16B*. Patients suffer spontaneous or provoked excessive bleeding that may be associated with morbidity and mortality. 

Studies of platelets revealed structural abnormalities like those found in gray platelet syndrome (GPS) characterized by severely reduced α granule numbers but increased δ granule numbers. Functional studies of platelets have seldom been performed. In one report [51], a platelet function study showed an absence of secondary aggregation wave with collagen and ADP but normal response to epinephrine and mildly decreased response to ristocetin. Another study [52] revealed reduced aggregation in response to AA, and no secondary aggregation wave with ADP. Further research is needed to determine the exact mechanism involved.

### 4.3. Defect in Response to a Single Activator

#### 4.3.1. Defect in Collagen-Induced Aggregation

This defect most often points to a hereditary [53,54,55] or acquired (autoantibodies) [56,57,58] deficiency in the GPVI, one of the main collagen receptors on the platelet surface. LTA in response to collagen can be completely absent or reduced in relation to the number of remaining receptors present on platelets. A peptide activator derived from collagen (collagen related peptide (CRP)) and convulxin (extracted from a snake venom) are selective GPVI agonists and may help to support the diagnosis orientation. Flow cytometry and Western blotting most often showed a platelet deficiency in GPVI.

Hereditary or acquired disorders related to signaling pathways downstream of GPVI are rare. Even so, some new targeted therapies used to treat malignant lymphoid or myeloid hemopathies such as Src, Syk or Btk inhibitors can inhibit collagen-induced aggregation by interfering with the GPVI pathway [59].

The toxin rhodocytine activates CLEC2 (C-type lectin-like receptor 2), whose signaling pathway resembles that induced by GPVI [60]. A defective response to rhodocytine, convulxin, and CRP can indicate signal failure downstream of these receptors, which all contain ITAM/hemi-ITAM motifs. Remarkably, platelets from patients carrying a Noonan syndrome (NS) displayed a significant reduction in LTA induced by low concentrations of GPVI and CLEC-2 activators, and a decrease in thrombus growth on a collagen surface under arterial shear stress. NS is an autosomal dominant genetic condition that affects one in 1000–2500 individuals—50% of patients with NS display pathological variants of the *PTPN11* gene coding a protein tyrosine phosphatase (SHP2) that is a critical regulator of signal transduction. Typical signs of NS include characteristic facial features, short stature, congenital heart defect, skeletal and thoracic anomalies, and developmental delay. Bleeding problems occur in 30–72% of patients. Recent data obtained in *PTPN11*-deficient mice support the hypothesis that defective GPVI proximal signaling is the cause of the reduction of NS platelet activation by collagen [61].

#### 4.3.2. Defect in Response to Ristocetin

Platelet disorders caused by von Willebrand factor receptor (Ib/V/IX complex) anomalies are accompanied by a specific defective response to ristocetin. Classically, the response to ristocetin is described not as aggregation but as agglutination in the presence of von Willebrand factor. However, the intensity of this response is clearly reduced or reversed by anti-IIb/IIIa agents indicating that ristocetin induces signaling and IIb/IIIa activation. Normal PRP does not respond to low doses of ristocetin (e.g., 0.5 mg/mL) but should respond to normal doses (e.g., 1 mg/mL). A reduced response to normal concentrations of ristocetin may indicate flaws in either von Willebrand factor or in the platelet GPIb/V/IX complex. Alternatively, enhanced response to ristocetin may evoke a 2B von Willebrand disease or a pseudo- (platelet-type) von Willebrand disease. Discrimination between these diseases is clinically important because they require different therapeutic management.

In Bernard and Soulier disease, which is a recessive inherited deficiency in the Ib/V/IX complex, aggregation in response to ristocetin (≥1.2 mg/mL) is reduced or absent. The diagnosis is suggested by the presence of macrocytic thrombocytopenia and is confirmed by demonstration of the defect by flow cytometry [62].

Pseudo- (platelet-type) von Willebrand disease is due to a mutation that increases the affinity of the Ib/V/IX complex for normal von Willebrand factor. LTA in response to a sub-threshold concentration of ristocetin (≤0.6 mg/mL) is present. Differential diagnosis from a 2B von Willebrand disease is established by positive cross-matching experiments (persistence of aggregation in response to low concentration of ristocetin of platelets from the patient combined with normal plasma in the case of pseudo- (platelet-type) von Willebrand [63]). Appropriate controls must be implemented to ensure the reliability of the results. Simplified procedures have been described for Ristocetin Induced Platelet Aggregation (RIPA) mixing experiments [64,65]. Although mixing experiments have been considered as the ‘gold-standard’ phenotypic test for provisional identification of pseudo- (platelet-type) von Willebrand disease, suspected cases have to be confirmed by genetic analysis whenever possible.

#### 4.3.3. The Special Case of Gαs Deficiency

An increased susceptibility to bleeding has been described in patients exhibiting hyperactive Gs signaling corresponding to a 36-base pair insertion in the gene encoding the extra-large Gs sub-unit [66]. The symptoms only appear if the mutated allele is inherited from the father. These cases are associated with syndromic clinical manifestations such as mental retardation, epilepsy, and moderately severe skeletal anomalies. LTA is normal, although bleeding time is significantly extended. The diagnosis is made by demonstrating increased inhibition of ADP-induced platelet aggregation by activators of receptors coupled to Gs such as PGI_2_ and PGE_1_.

## 5. Hereditary Platelet Disorders with No Molecular Cause Characterized Yet 

### 5.1. Isolated Defect in Response to Epinephrine

A defective response to epinephrine (5–10 μM) may occur in healthy subjects. It has been reported in 16% of healthy Japanese persons and related to a reduced number of epinephrine receptors (alpha 2-adrenergic, α2A) at the platelet surface [67]. This has also been described in the Quebec platelet disorder [68] where platelets failed to aggregate in response to 6–10 µmol/L epinephrine despite normal numbers of platelet α2A receptors.

### 5.2. Suspicion of Gαq Deficiency

Gαq (Figure 1) is downstream of the P2Y_1_, TXA_2_ and PAR receptors. A 25% deficiency in Gαq has been demonstrated in one family, but to date it has not been associated with any molecular abnormality [69]. Platelet exploration showed impaired activator-induced LTA, secretion, arachidonate release, and Ca^2+^ mobilization. A profound defective response to epinephrine (8 μM) and collagen (2.5 μg/mL) without any abnormality in the response to exogenous AA was reported. The initial hypothesis was a reduced AA release, but no molecular abnormality has yet been identified to support this hypothesis. This patient was therefore presumed to have a Gαq protein deficiency. Immunoblot analysis of Gα subunits in the patient’s platelet membranes showed a decrease in Gαq (<50%) but not Gαi, Gαz, Gα12, and Gα13 [70]. Decreased expression of the Gαq coding gene *GNQA* was not observed in neutrophils, suggesting a hematopoietic lineage-specific defect [71].

### 5.3. Suspicion of Phospholipase Cβ defect

This defect was proposed by Rao in 1989. Abnormal calcium metabolism and a decrease in the level of phospholipase Cβ2 mRNA [72] in platelets, but not in neutrophils, were demonstrated. In these patients, LTA is reduced in response to most activators. The profound decrease in collagen-induced aggregation may be linked to a reduced amplification by TXA_2_. No molecular abnormality has been identified so far to support a constitutional origin.

### 5.4. Defect Affecting the P2X1 Receptor

The P2X1 receptor, whose agonist is ATP, helps regulate calcium influx during platelet activation. A patient with severe bleeding diathesis associated with a naturally occurring dominant negative *P2X1* mutant has been described. A selective defect in ADP-induced aggregation was observed without any abnormalities in response to AA, TRAP14, or ristocetin [73]. However, the relationship between genotype and phenotype in the patient described is unclear, because the P2X1 receptor, which is activated by ATP, has no role in ADP-induced platelet aggregation [74]. 

### 5.5. Defect Affecting GPIa

Platelet GPIa/IIa, is an important mediator of platelet adhesion to fibrillar collagens at sites of vascular injury. GPIa deficiency associated with the absence of platelet aggregation in response to collagen has been observed in patients with bleeding symptoms [75,76], suggesting a causal relationship between bleeding and GPIa deficiency. However, these cases did not benefit from GPVI exploration. In addition, the surface GPIa/IIa receptor density varied considerably between individuals from about 1800 to 800 molecules per platelet, which can hinder interpretation of the decrease in GPIa expression [77]. Acquired GPIa/IIa defects have also been described and were associated [76] or not [78] with bleeding events. Overall, the involvement of GPIa in bleeding disorders needs further investigation.

## 6. LTA Versus Other More Recently Developed Methods

In the 1960s, LTA was the primary method used to assess platelet function. Interestingly, this test was still the predominant test used for the diagnosis of platelet function disorders in 2014. In that year, the ISTH/SSC published a major study to gain a better understanding of diagnostic practices for constitutional platelet function disorders worldwide [79]. A 67-item questionnaire was distributed to ISTH members and members of several national hemostasis and thrombosis societies. Two hundred and two laboratories from 37 countries participated in the survey. For most sites, the first functional screening tests were LTA and PFA-100^®^ (Platelet Function Analyser, Dade Berhing, Marburg, Germany). Under the hemorheological conditions of the PFA-100 realization, von Willebrand factor becomes the principal mediator of platelet aggregation. This device is therefore very sensitive to von Willebrand factor abnormalities. The occlusion time measured by the PFA-100 becomes longer in cases of low hematocrit or thrombocytopenia. The effects are pronounced for a value < 100 × 10^9^/L. Therefore, it is not possible to assert the existence of platelet pathology from the PFA-100 results when interpreted alone. Many other laboratories used platelet aggregation associated with luminescence (21.3%) and/or impedance aggregation (11.7%) as first steps. Tests used as a second step were most often flow cytometry (64.7%) or molecular genetic analysis (36.4%).

Since the late 1980s, the evaluation of platelet (dys)function has become increasingly necessary in a variety of clinical settings, and other methods such as whole-blood platelet aggregation have become available. These methods are based on different measurement principles and some of them have been developed for rapid bedside management, especially in intensive care units. The key feature of whole-blood-based methods is that platelet function is assessed under more physiological conditions, as contributions from other blood components can also affect platelet function. In addition, these methods use a small amount of blood in which all subpopulations of platelets are present with no manipulation of the sample without platelet activation, allowing rapid analysis of platelet function [80]. Despite some advantages, some of these devices are not flexible, not widely available, and influenced by confounding factors such as platelet counts [81]. In addition, their performance in diagnosing constitutional platelet pathologies is poorly documented. Further studies are needed to evaluate their potential role in the diagnosis of inherited platelet dysfunction.

## 7. Conclusions

For more than forty years, LTA has been essential in platelet exploration. LTA is the most widely used method for detecting platelet function disorders. Using this test, several etiologies of platelet dysfunction have been recognized and characterized. It is a flexible method, and a considerable amount of data can be obtained on the different pathways of platelet activation. Although LTA is accepted as the gold standard test for diagnosing platelet function disorders, there are some problems. The technique can be affected by different pre-analytical conditions (type of anticoagulant, lipid plasma, hemolysis, or low platelet count), and by different procedural conditions (requires fresh samples, manual preparation of PRP, use of a large volume of blood, and different concentrations of different activators, lack of standardization and quality controls, etc.). In addition, laboratory staff must have a high degree of skill, experience, and expertise to carry out the exploration and interpret the results. 

Faced with the current diversity of platelet dysfunctions, it is important to optimize the interpretation of platelet aggregation results by using a well-adapted strategy. A screening test including aggregation induced by 2 and 10 μM ADP, 5 μM epinephrine, 2 and 10 μg/mL collagen, 10 and 50 μM TRAP, and 1 mM AA can provide an initial indication of the platelet impairment (Table 1). A second round of testing could include high concentrations of ADP (100 μM), stimulation with the TXA_2_ analog, U46619, convulxin, PMA etc., depending on the suspected diagnosis. ADP 100µM can be useful to diagnose complete P2Y_12_ deficiencies, stimulation with the TXA_2_ analog, U46619, can allow the TP receptor to be distinguished from TXA_2_ synthesis deficiencies, convulxin can be used as a GPVI specific activator, and PMA can check the correct functioning of the PKC pathway. Although the use of different activators and different concentrations of these activators often helps to arrive at a valuable diagnostic hypothesis, in some cases this strategy has insufficient discriminating power. Because platelet dysfunction can be caused by a plethora of factors, to make a diagnostic hypothesis, the results obtained by LTA must be supplemented by other analytical tests (e.g., flow cytometry analysis for identification and quantification of specific platelet components, assessment of platelet secretion, and specific assays for platelet compounds).

Given that LTA results still have many limitations and remain difficult to interpret in a significant proportion of cases, their place in the workflow of diagnosis of hereditary platelet disorders needs revisiting. Next generation sequencing (NGS) technology has given rise to the rapid identification of genes associated with platelet disorders. It has already vastly helped to improve and expand molecular diagnosis and knowledge of platelet pathophysiology. Identification of a genetic basis for platelet disorders discovered using NGS will drive changes in diagnosis strategy. As further research is done on platelet disorders and on the identification of the causal variants associated with the disease, NGS will soon be fully integrated into the diagnostic setting, providing a wider range of diagnostic options for clinicians to use. It would be helpful if further research could identify the best strategy from a socioeconomic point of view. 

From its present use as a first-line screening test, LTA could move on to become a second-line functional test for confirmation of genetic variations identified by high-throughput sequencing techniques.

## Figures and Tables

**Table 1:** Main diagnosis orientations after first-line LTA testing using different activators at low (2 μM ADP, 2 μg/mL collagen, and 10 μM TRAP), and high concentrations (10 μM ADP, 5 μM epinephrine, 10 μg/mL collagen, 50 μM TRAP, 1.2 mg/mL ristocetin, and 1 mM AA).

Absence of Aggregation in Response to Multiple Activators Except Ristocetin	
LTA Traces	Diagnostic Orientation
Absence or marked reduction of aggregation in response to low and high agonist concentrations	If quantitative deficiency in GPIIb/IIIa: Glanzmann thrombasthenia
Absence or marked reduction of aggregation in response to low and high agonist concentrations	If qualitative deficiency in GPIIb/IIIa: Glanzmann thrombasthenia variant
	If immune deficiency (infections hyperleukocytosis): Type III LAD
**Reduced aggregation in response to multiple activators**	
Absence of response to low concentrations, but normal or improved response with high concentrations of activators except for epinephrine. Reduced aggregation responses to ADP (2-10 µM) and epinephrine (5–25 µM) in all reported cases is a consistent laboratory phenotype.	CalDAG-GEFI deficiency
Reduced response to ADP even at high concentrations (100 μM); reduced (or reversible) response to low concentrations and improved response to high concentrations with the other activators.	Severe homozygous P2Y_12_ deficiency
Reduced (or reversible) response to low concentrations of activators and improved response with high concentrations of the activators. The defective response to 10 µM ADP is often moderate.	Severe dense granule defects
Absence or reduced response to AA and reduced (or reversible) response to the other activators such as epinephrine and ADP. The responses improve on increasing activator concentrations.	TXA_2_ pathway disorder. The use of a specific TP agonist distinguishes between defect in TP receptor or TXA_2_ synthesis
**Defective response to a specific activator**	
Reduced response to collagen	GPVI disorder. The use of more specific GPVI agonist may help to confirm the diagnosis orientation.
Reduced response to normal or low concentration of ristocetin.	Defect in the GPIb/vWF axis.

LAD: leukocyte adhesion deficiency; LTA: light transmission aggregometry; TXA_2_: thromboxane A_2_; TRAP: thrombin receptor-activating peptides.
